# Adult facilitation becomes competition as juvenile soapberry bugs age

**DOI:** 10.1002/ece3.8056

**Published:** 2021-09-08

**Authors:** Meredith Cenzer

**Affiliations:** ^1^ Department of Ecology and Evolution The University of Chicago Chicago Illinois USA

**Keywords:** intraspecific interactions, niche shift, ontogeny, plant defense, stress

## Abstract

Intraspecific interactions can change from facilitative to competitive depending on the organism's ontogeny. In plant‐feeding insects, host plant defenses can be strengthened or weakened by insect feeding and can therefore be important for determining whether two insects feeding on the same plant help or harm each other's fitness. Here, I conducted two experiments looking at the direct effect of a physical seed defense and the role of intraspecific facilitation in reducing the effects of that defense for juveniles of the red‐shouldered soapberry bug. I demonstrate that juveniles are severely inhibited by the tough seed coat of their host plant, leading to high mortality early in development. Adults, in contrast, can create holes through which younger individuals could potentially feed. I manipulated whether or not seeds were fed on by adults on two host plant species: a well‐defended native host and a poorly defended introduced host. Survival in the first week of development was dramatically improved by prior adult feeding, and this facilitation was stronger on the well‐defended host plant. However, the benefits of prior adult feeding ceased after the first week of development and shifted to having a negative effect on survival, development time, and body size. These results indicate that ontogeny is a key factor determining the effects of plant defenses and the strength and direction of intraspecific interactions across multiple host plant species.

## Graphical abstract

I demonstrate experimentally that adult soapberry bugs facilitate early juvenile survival by breaking through the tough seed coat of their host plant, but that prior adult feeding shifts to having a negative impact on fitness late in juvenile development. I show that the positive effects of facilitation are more beneficial on the well‐defended native host plant than on the wimpier introduced host.

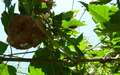

## 1 INTRODUCTION

Ecological interactions can be defined as positive, negative, or neutral based on how individuals or species impact each other's lifetime fitness. In a classic competitive interaction, two individuals who share the same resource negatively impact each other's survival and/or fecundity by reducing availability or quality of that resource directly or indirectly (e.g., Crombie, 1945; Tilman, 1987). Conversely, two individuals can positively influence each other by making resources easier to access, thus increasing at least one individual's fitness in a facilitative interaction (Bronstein, 2009; McIntire & Fajardo, 2014). Defining interactions based on their lifetime impacts on average fitness has been foundational for understanding behavior, population dynamics, and persistence (e.g., Crombie, 1945). However, looking at the average lifetime impact or at a single life stage may miss much of the complexity of how individual interactions change over time (Boege & Marquis, 2005; Yang & Rudolf, 2010).

As individuals age, they undergo quantitative and qualitative changes that can alter how they interact with other members of their community (Boege & Marquis, 2005; Boone et al., 2002; Magalhaes et al., 2005; Wilbur, 1980; Wissinger, 1992). In many systems, one species can shift between having positive and negative effects on another species over ontogeny; for example, insects that are herbivores as juveniles may become pollinators as adults (Adler & Bronstein, 2004; Alarcón et al., 2008; Bronstein et al., 2009) and “nurse plants” that shelter young seedlings from harsh abiotic conditions become competitors as those seedlings age (Callaway & Walker, 1997; Carrión et al., 2017; Holzapfel & Mahall, 1999; Miriti, 2006). These shifts from positive to negative impacts on fitness (or vice versa) can explain behavioral or spatial associations that vary in time and space that cannot be explained by the impact on lifetime fitness alone. For example, in sedentary species, interactions that begin as facilitative create spatial associations (Callaway, 1995) that cannot be avoided by movement if they later become negative. In mobile species, positive to negative shifts across ontogeny may create behavioral shifts from aggregative to solitary behavior (as suggested in Denno & Benrey, 1997). These ontogenetic shifts may create or resolve conflicts between age classes. For example, if early life stages benefit from the presence of conspecifics, they may actively seek associations with older conspecifics. If the interaction becomes negative as they age, however, older individuals may avoid the company of younger conspecifics in favor of solitude. In contrast, shifts from negative to neutral effects may resolve conflict, as in ontogenetic niche shifts where adults use different resources from juveniles (Pyrzanowski et al., 2021; Sánchez‐Hernández et al., 2019).

Whether an individual interaction has positive or negative effects on fitness can be highly sensitive to environmental conditions (e.g., Beduschi & Castellani, 2013; le Roux et al., 2013). In plants, the Stress Gradient Hypothesis proposes that pairwise interactions are more facilitative when abiotic stress is high, and shift to being more competitive as stress decreases (Grime, 1973; Gomez‐Aparicio et al., 2004; Sthultz et al., 2006; He et al., 2013; but see Maestre et al., 2005). When stress is low, most individuals are capable of persisting in an environment, but success is driven by how well an individual or species extracts resources compared with the other individuals or species present. As environments become increasingly stressful, fewer individuals or species are capable of persisting, and competition therefore weakens. Under these conditions, success may then be determined by the ability of individuals to tolerate stress. Stress‐tolerant individuals can modify local conditions, which can then facilitate the persistence of other, less stress‐tolerant individuals. While the Stress Gradient Hypothesis was designed to explain spatial patterns, the same logic can be applied to stress changing over time (e.g., Leverett, 2017). In particular, ontogenetic shifts from facilitation to competition can occur in systems where juveniles have a low capacity to deal with harsh conditions, but individuals become more stress tolerant as they age (e.g., Miriti, 2006). While the environment remains constant, its relative stressfulness decreases from the perspective of the individual. Thus, we might expect young juveniles to benefit most from facilitation, but experience increasingly competitive interactions as they age (Callaway & Walker, 1997; Holzapfel & Mahall, 1999; Miriti, 2006). Even when negative effects of competition are costly, the positive effects at one life stage may permit persistence in environments that would otherwise be unusable.

Early in development, insect herbivores can be highly susceptible to mortality as a result of host plant defenses (Cenzer, 2016b; Yang et al., 2020; Zalucki, Brower, et al., 2001; Zalucki, Malcolm, et al., 2001). The intensity of plant defenses may be reduced by heterospecifics feeding on the same plant (Huang et al., 2013; Kaplan et al., 2008; Soler et al., 2012) or by conspecific groups, which can overcome defenses that would prevent individuals from feeding alone (Kawasaki et al., 2009; Raffa & Berryman, 1983). Conversely, plant defenses may intensify competitive interactions if herbivory induces increased expression of defensive compounds or structures (Karban & Baldwin, 1997; Quintero & Bowers, 2013; Van Zandt & Agrawal, 2004) or if reduced plant quality increases individual resource demand through compensatory feeding (Simpson & Simpson, 1990). These interactions need not occur synchronously in time, as the induction or suppression of plant defenses by herbivores can persist well beyond the moment of feeding (Benevenuto et al., 2020; Bouagga et al., 2018; Kant et al., 2015; Nosko & Embury, 2018).

Studies of gregarious feeding behavior in caterpillars indicate that group feeding is beneficial during early development in some species (Denno & Benrey, 1997; Fiorentino et al., 2014; Fordyce, 2003; Inouye & Johnson, 2005). Aggregations typically break up in later developmental stages (Denno & Benrey, 1997; Fordyce, 2003; Inouye & Johnson, 2005), potentially as a result of increasing resource demand in older juveniles. This pattern suggests that competition becomes a more important force than facilitation as juvenile ontogeny progresses and that plant defenses may play an important role, particularly in the earliest stages of development.

In this study, I use a simple physical plant defense to address four general questions. First, how does intraspecific feeding influence juvenile performance? Second, does this effect vary across ontogeny? Third, what is the physical mechanism mediating intraspecific facilitation (and/or competition) and can we recreate its effects in the absence of intraspecific feeding? Finally, does the effect of intraspecific feeding differ between a well‐defended and a poorly defended host plant?

### 1.1 Study system

The red‐shouldered soapberry bug *Jadera haematoloma* (Hemiptera: Rhopalidae) is a seed‐feeding specialist on plants in the Sapindaceae. In the field, adults and juveniles form mixed aggregations of tens to thousands of individuals on and around their host plants, in which both feeding and mating occur (Carroll, 1988), creating the potential for both facilitation and competition. At all life stages, individuals feed by drilling a hole through the seed coat with their rostrum, “spitting” digestive juices into it, and ingesting the partially liquified developing plant through the hole using a thread‐like stylet. When reared in isolation, juveniles suffer high mortality early in development (Cenzer, 2016b) and typically die before doing any visible damage to the seed, suggesting they are unable to circumvent one or more of the seed's defenses at this stage. Adults and older juveniles, in contrast, have relatively low mortality (Cenzer, 2016a) and often attempt to drag seeds away from conspecifics up nearby plant stems (personal observation).

**FIGURE 1:**
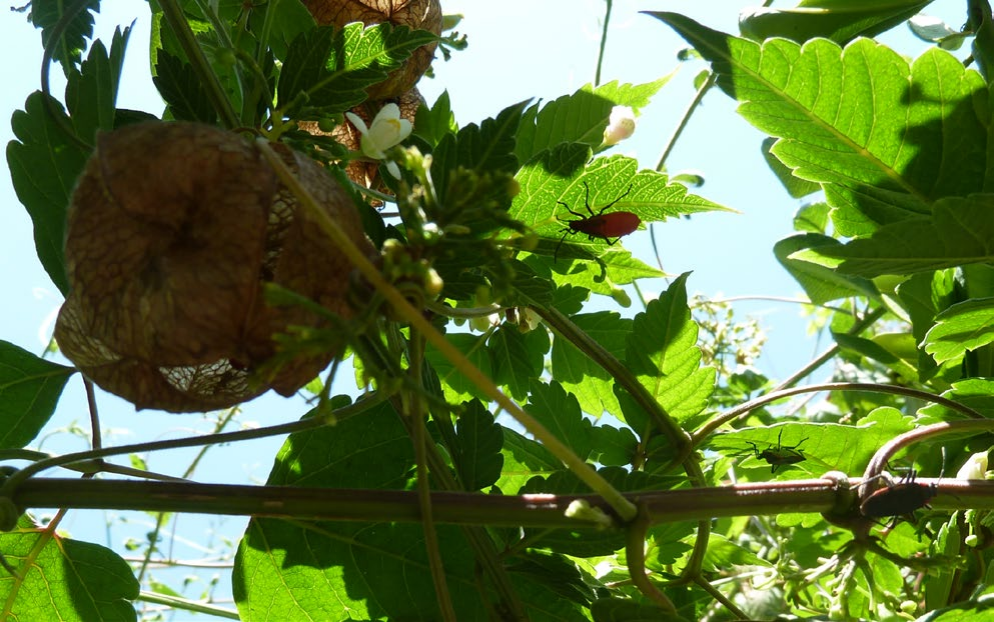
Fifth instar juvenile (top center) and adults (lower right) of the red‐shouldered soapberry bug near dehisced seedpods (center left) on the native host, *Cardiospermum corindum*

In Florida, *J*. *haematoloma* has two primary host plants that differ in the strength of their seed defenses: the native balloon vine (*Cardiospermum corindum*) and the introduced golden rain tree (*Koelreuteria elegans*). *Cardiospermum corindum* has seeds that are defended by an inflated seedpod that is too large for juveniles to feed through. Therefore, juveniles must wait for seedpods to open (dehisce) before feeding, at which point adults have often already done damage to the seeds (Figure 1). Juveniles must then contend with a tough, hardened seed coat. Juveniles reared on undamaged, dehisced seeds of this host have very high mortality during the first week of development (Cenzer, 2016b). In contrast, the introduced host plant *K*. *elegans* has open seedpods and relatively soft seeds that are low in chemical defenses (Umadevi & Daniel, 1991), and juvenile survival on *K*. *elegans* is quite high even when juveniles are reared alone (Cenzer, 2016a). Populations of soapberry bugs living on these two host plants have a history of local adaptation in morphology, development time, and juvenile survival (Carroll et al., 1997; Carroll & Loye, 1987); however, recent gene flow has shifted all populations toward higher fitness on the introduced *K*. *elegans* and lower fitness on the native *C*. *corindum* (Cenzer, 2016a). While seedpod size is known to be an important agent of natural selection on adult beak length in this system (Carroll & Boyd, 1992; Cenzer, 2017), it is unknown whether seed coat toughness may act as a selective agent on adult or juvenile feeding morphology.

The difference in mortality paired with the difference in seed coat toughness suggests the physical barrier of the seed coat itself is a good candidate as the primary defense stopping young juveniles from feeding. On both hosts, adult feeding leaves visible holes in seeds that may serve as access points for young individuals, allowing them to circumvent this defense when feeding after adults. However, these access points may come at the cost of nutrients being reduced or altered by individuals who fed earlier.

Finally, the difference in mortality over time and between hosts gives us two predictions derived from the Stress Gradient Hypothesis. First, facilitation is likely to be more important on the more stressful native host plant (*C*. *corindum*) than on the more benign introduced host plant (*K*. *elegans*). Second, younger juveniles experience greater relative stress (as indicated by higher mortality) than older juveniles, suggesting facilitation is likely to be more important early in ontogeny than late in ontogeny.

Using the red‐shouldered soapberry and its two host plants in Florida, I test four specific hypotheses:

H 1 Adults have a positive effect on juvenile survival early in development, when juvenile survival is lowest, by creating physical entry points in the seed coat of host plant seeds.

H 2 As juveniles age, the effect of prior adult feeding on fitness becomes neutral or negative.

H 3 Manually cracking the physical barrier of the seed coat mimics the positive effects of adult feeding on early juvenile success.

H 4 The benefits of adult feeding and of cracking the seed coat are stronger on the well‐defended native host plant than on the poorly defended introduced host.

## 2 METHODS

I conducted two experiments to test these hypotheses. In Experiment 1, I tested the effects of prior adult feeding on juvenile success across ontogeny (H1, H2) on two host plants (H4). In Experiment 2, I tested whether physically breaking through the seed coat mimics the effects of adult facilitation (H3) on two host plants (H4). Both of these experiments were conducted with seeds removed from the seedpod.

### 2.1 Collection

I collected adult *J*. *haematoloma* for Experiment 1 in March–April 2015 from four locations in Florida, two from each host plant (Leesburg [*K*. *elegans*], Lake Wales [*K*. *elegans*], Key Largo [*C*. *corindum*], Plantation Key [*C*. *corindum*]) and one location in California (Davis [*Koelreuteria paniculata*]) (Appendix S1). To test the direct effects of the physical seed coat barrier (Experiment 2), I collected soapberry bugs in April 2014 from 7 locations in Florida: Gainesville [*K*. *elegans*], Leesburg [*K*. *elegans*], Lake Wales [*K*. *elegans*], Ft. Myers [*K*. *elegans*], Homestead [*K*. *elegans*], Homestead [*C*. *corindum*], Key Largo [*C*. *corindum*], and Plantation Key [*C*. *corindum*]. I collected host plant seeds from each Florida site in December 2013, April 2014, and April 2015 and stored them at 4°C until they were used for rearing. Seeds were only collected from *K*. *paniculata* in April 2015. Seeds with visible indications of previous feeding were discarded. I tested all seeds for viability by placing them in water and discarding seeds that floated. I collected from 5 to 10 individual trees at each *K*. *elegans* site, 3–15 individual vines at each *C*. *corindum* site, and from 6 trees at the *K*. *paniculata* site.

### 2.2 Experiment 1: interactions between adults and juveniles

The first experiment tested the effects of prior feeding by adults on juvenile performance (H1, H2, H4). The two seed treatments were intact seeds and seeds that had been fed on by adults. For this experiment, I used juveniles from the first laboratory generation descended from the 2015 field collection. Juveniles were assigned to one of two rearing host treatments (*C*. *corindum* or *K*. *elegans*) and one of two seed treatments (intact or prior feeding) in a full factorial design (*N* = 71, 71, 71, 70). For consistency and convenience, I created the prior feeding treatment using local bugs from Davis, California, where the primary host plant is *K*. *paniculata*. To confirm that there was nothing unusual about this population or host, I also conducted this experiment using *K*. *paniculata* as both the rearing host (Appendix S1) and with juveniles from that field collection host (Appendix S2) at reduced sample sizes.

All rearing was carried out in controlled environmental chambers (Sanyo Versatile Environmental Test Chambers, Sanyo Electric Co, Osaka, Japan) at 28°C during the day and 27.5°C at night, 50% relative humidity with a 14:10 light:dark cycle, following spring climate conditions in the field and those used in earlier work (Carroll & Boyd, 1992; Cenzer, 2016a). Adults collected from the field were housed as mating pairs in vented Petri dishes lined with filter paper and given water in a microcentrifuge tube stoppered with cotton (“water pick”) and 3 seeds of their field host plant. When adult pairs began producing eggs, they were collected daily until hatching.

To produce both seed treatments, seeds were first soaked for 18 hr in deionized water. I set up vented Petri dishes lined with filter paper, a water pick, and two seeds of different species secured in place using modeling clay. The prior feeding treatment was produced by adding five adults of mixed sex to each dish. These adults were collected in Davis, CA, from *K*. *paniculata* (Appendix S1) and held for 24 hr without food to encourage feeding. Petri dishes were then returned to the incubator and remained for an average of 55 hr before being used for rearing. This method was chosen after trial and error with different seed numbers, adult numbers, starvation periods, soaked and unsoaked seeds, and seed species configurations with the objective of creating uniform damage across seeds. This method resulted in 94.7%–98.9% of seeds assigned to the prior feeding treatment receiving visible feeding damage. The hypothesized method of facilitation by adults is the damage they create on the seed; therefore, seeds assigned to the prior feeding treatment that showed no visible signs of damage were not used in the experiment. This exclusion may have created a bias such that very poor‐quality seeds might not have been used in the prior feeding treatment due to filtering by adult choice. Such a bias could result in a higher average quality in the prior feeding than in the intact treatment; because rates of nonfeeding were very low, however, such effects would be small. This method was chosen, rather than housing adults and juveniles together to feed simultaneously, for three main reasons. First, for the native host, seeds have often experienced adult feeding prior to becoming available to juveniles. Second, soapberry bugs will cannibalize each other at high rates in captivity, particularly during molting, making shared housing intractable. Finally, I chose to use a truncated amount of adult feeding to keep seeds from becoming quickly exhausted and to allow the exploration of the relationship between a fixed number of holes in the seed and juvenile fitness.

To assess juvenile fitness, I measured daily survival, development time to adulthood, and final adult body size. Nymphs were isolated within 12 hr of hatching to reduce egg cannibalism and housed individually. Nymphs were reared in mesh‐lidded portion cups, lined with filter paper, and containing a water pick and a seed of their assigned rearing host treatment. Juveniles were distributed in a split‐brood cross‐rearing design, such that full siblings from all families were represented in all treatments. Before I introduced juveniles to the prior feeding treatment, I recorded the number of holes adults had drilled in each seed. After the “early” developmental period (7 days after hatching, following completion of the first instar), I added additional seeds (a total of two for *K*. *elegans* and three for *C*. *corindum*, for a total seed mass of ∼150 mg) to each juvenile's container, after again recording the number of holes created by adults in the prior feeding treatment. Individual containers were rotated daily within the growth chamber. Water, filter paper, and cotton were changed weekly. Nymph survival and whether or not they had reached adulthood were assessed daily. Upon reaching adulthood, bugs were allowed 1 day for the exoskeleton to harden and were then frozen at −20°C for morphological analyses.

All analyses were conducted in R version 3.4.2 (R Core Team, 2014). The sets of models evaluated for each response variable are discussed in greater detail below. For each response variable, all models were compared using Akaike information criterion (AIC), a metric combining goodness of fit and model simplicity to identify models that minimize information loss. I used AIC to calculate the relative likelihood of each model in the set, compared to the model with the lowest AIC. All models that were >5% as probable as the lowest AIC model to minimize information loss were examined. If effects were not significant and in the same direction in all examined models, then models were directly compared using chi‐squared tests; if two models were not significantly different based on this test, the simpler of the two models was chosen as the best available representation of the data. In all cases reported here, the specific test statistics and effect sizes in the results section were taken from the model with the highest relative likelihood. The highest performing model for each response variable in Experiment 1 is reported in Table 1.

**TABLE 1:** Top models for response variables in Experiment 1

Response variable	Top model
Total survival	Survival ~ rearing host + seed treatment + field host
Early survival	Early survival ~ rearing host * seed treatment + field host
Early survival (prior adult feeding treatment only)	Early survival ~ hole number
Late survival	Late survival ~ rearing host * seed treatment + field host
Late survival (prior adult feeding treatment only)	Late survival ~ rearing host + hole number + field host
Development time	Development time ~ seed treatment + field host
Development time (prior adult feeding treatment only)	Development time ~ rearing host + hole number + field host
Thorax width	Thorax width ~ rearing host + seed treatment * sex
Thorax width (prior adult feeding treatment only)	Thorax width ~ rearing host + hole number + sex

I evaluated the effects of adult feeding on juvenile performance (H1 & H2) by including the seed treatment (intact or prior feeding) as a fixed factor in the analyses of survival, development time, and final adult body size. To evaluate whether the effects of the seed treatment on survival differed early and late in development, I separately evaluated survival during the first week of development (“early survival”) and after the addition of new seeds on day 7 (“late survival”). To address the hypothesis that the effects of adult feeding would differ between host plants (H4), I included the main effect of rearing host and the rearing host * seed treatment interaction in the analyses of all response variables. Populations from different field collection hosts have sometimes shown differences in development time and adult morphology; therefore, field host was included as a main effect in all models and in interactions when possible. Sex (which can only be determined in adults) has also been shown to impact development time and adult morphology in previous studies; therefore, sex was also included as a main effect and in interactions when sample size allows (Tables 1 and 2).

For total and early survival, all possible models including the main effects of rearing host (*C*. *corindum* or *K*. *elegans*), field collection host (*C*. *corindum* or *K*. *elegans*), seed treatment (intact or prior feeding), and all possible two‐way interactions were considered. These models were also analyzed as generalized linear mixed models with the random factor of individual population nested within ancestral host and the random factor of family nested within individual population. Survival was modeled using a binomial error distribution with a logit link.

**FIGURE 2:**
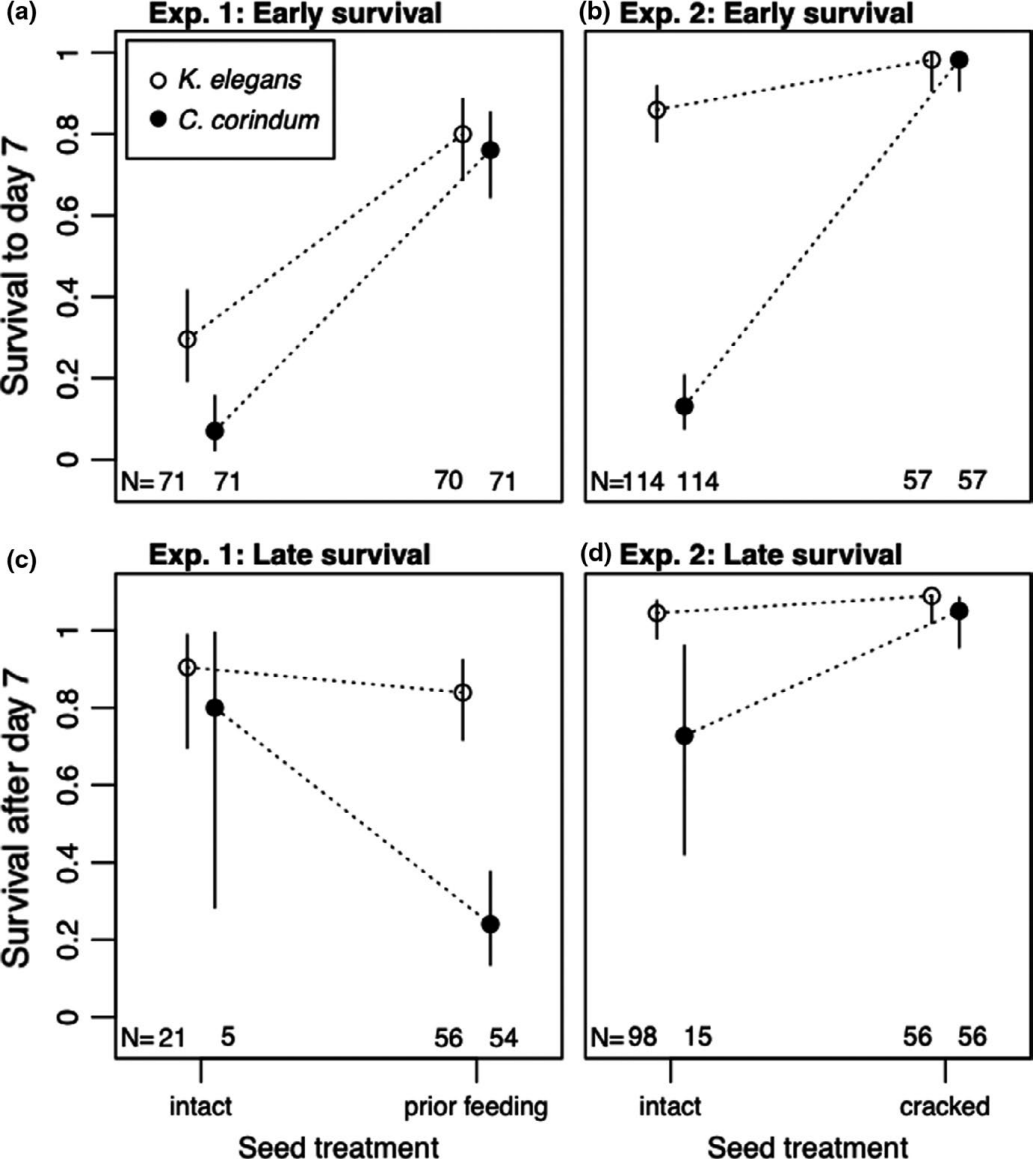
Soapberry bug juvenile survival prior to day 7 in Experiments 1 (a) and 2 (b) and juvenile survival after day 7 in Experiments 1 (c) and 2 (d). Open circles represent bugs reared on *Koelreuteria elegans,* and filled circles represent bugs reared on *Cardiospermum corindum*. Points are means, and error bars are 95% binomial confidence intervals computed using the Pearson‐Klopper method

The response variables late survival, development time, and body size had reduced sample sizes in some treatments due to strong treatment effects on survival (see Figures 2c, 4a, and 5a for corresponding sample sizes). Therefore, I considered a reduced set of models with fewer possible interactions for these variables, and without the random factors family and population. For late survival, I compared all models with the main effects of seed treatment, rearing host, field collection host, and the rearing host * seed treatment interaction. As with early and total survival, I used a binomial error distribution with a logit link.

For development time, I again compared all models with the main effects of seed treatment, rearing host, field collection host, and the rearing host * seed treatment interaction, and the additional main effect of sex. In these models, I used a Gamma error distribution. Gamma distributions are positive definite with skew to the right; they are flexible distributions commonly used in modeling waiting times.

Body size is highly dimorphic between sexes, which may result in different reactions to treatments between sexes as they preferentially invest in different body parts. For body size, I again compared all models with the main effects of seed treatment, rearing host, field collection host, sex, and the rearing host * seed treatment interaction, with the addition of interactions between sex * seed treatment, sex * rearing host, and sex * field collection host. I modeled body size with a Gaussian error distribution and tested residuals for normality using Shapiro–Wilk normality tests.

In order to address the hypothesis that changes in survival, development time, and body size were the result of physically breaking through the seed coat (H1), I conducted a second set of analyses to test the effect of the number of holes drilled by adults on each of these response variables. Analyses of hole number were run only on data from the prior feeding treatment to distinguish the main treatment effect from the effect of hole number. This effect was evaluated using the same models that were considered for each response variable on the full dataset, but replacing seed treatment with hole number.

It should be noted that variation in hole number was produced by adult feeding decisions rather than by direct manipulation. Therefore, analyses of hole number may be testing the effect of some underlying variable that influenced the number of holes adults chose to drill in each seed (e.g., seed toughness) rather than the direct effect of hole number itself.

### 2.3 Experiment 2: manually cracking the seed coat

The second experiment directly tested the effects of the seed coat on juvenile performance to address the hypothesis that juvenile success can be facilitated by circumventing the physical barrier of the seed coat (H3) on two host plants (H4). Juvenile rearing was carried out following the same methods described above in Experiment 1. For this experiment, I used the second laboratory generation of juveniles descended from the April 2014 field collection. The first laboratory generation was used for experiments described in (Cenzer, 2016a).

As in Experiment 1, juveniles were distributed in a split‐brood cross‐rearing design, such that full siblings from all families were represented in all treatments. Upon hatching, juveniles were randomly assigned to a rearing host (either *C*. *corindum* or *K*. *elegans*) and a seed treatment (intact or cracked seed coat) (*N* = 114, 114, 57, 57). Sample sizes in the intact seed treatments were deliberately higher to increase power for estimating late survival, development time, and body size. I administered the cracked seed treatment by gently clamping seeds in pliers and tightening the pliers just until a crack formed in the seed coat. As above, daily survival, development time, sex, and final body size were recorded for each individual.

The effect of the seed coat on juvenile performance (H3) was evaluated by including the seed treatment (intact or cracked) as a fixed factor in the analyses of survival, development time, and final adult body size. To evaluate whether the effects of the seed treatment on survival differed early and late in development, I again separately evaluated survival in the first week of development (“early survival”) and survival after the addition of new seeds on day 7 (“late survival”). As in Experiment 1, the hypothesis that treatment effects differ between host plants (H4) was evaluated by including the rearing host main effect and the rearing host * seed treatment interaction. Model comparison was made using AIC, as in Experiment 1. Top models for each response variable are reported in Table 2.

**TABLE 2:** Top models for response variables in Experiment 2

Response variable	Top model
Total survival	Survival ~ rearing host * seed treatment
Early survival	Early survival ~ rearing host * seed treatment
Late survival	Late survival ~ rearing host + seed treatment + field host
Development time	Development time ~ rearing host + seed treatment + field host + sex
Body size	Thorax width ~ rearing host * sex

For total, early, and late survival, all possible models including the main effects of rearing host (*C*. *corindum* or *K*. *elegans*), field collection host (*C*. *corindum* or *K*. *elegans*), seed treatment (cracked or intact), and all possible two‐way interactions were considered. These models were also analyzed as generalized linear mixed models with the random factor of individual population nested within ancestral host and the random factor of family nested within individual population.

In spite of higher starting sample sizes, the response variables development time and body size again had reduced sample sizes as a result of mortality (surviving *N* = 94, 10, 56, 54). Therefore, I again considered a reduced set of models with fewer possible interactions for these variables and without the random factors family and population. For development time, I compared all models with the main effects of seed treatment, rearing host, field collection host, and sex, as well as the rearing host * seed treatment interaction. I again used a Gamma error distribution for development time. Two extreme outliers, that fell about 10 standard deviations above the mean and 6 standard deviations above the next highest measurement, were dropped from the analyses of development time.

For body size, I compared all models with the main effects of seed treatment, rearing host, field collection host, and sex, as well as the rearing host * seed treatment interaction. To account for possible effect changes due to the sexual dimorphism in this species, I also included the three possible pairwise interactions with sex: sex * seed treatment, sex * rearing host, and sex * field collection host.

## 3 RESULTS

### 3.1 (H 1) Early in development, prior adult feeding facilitates juvenile survival

**FIGURE 3:**
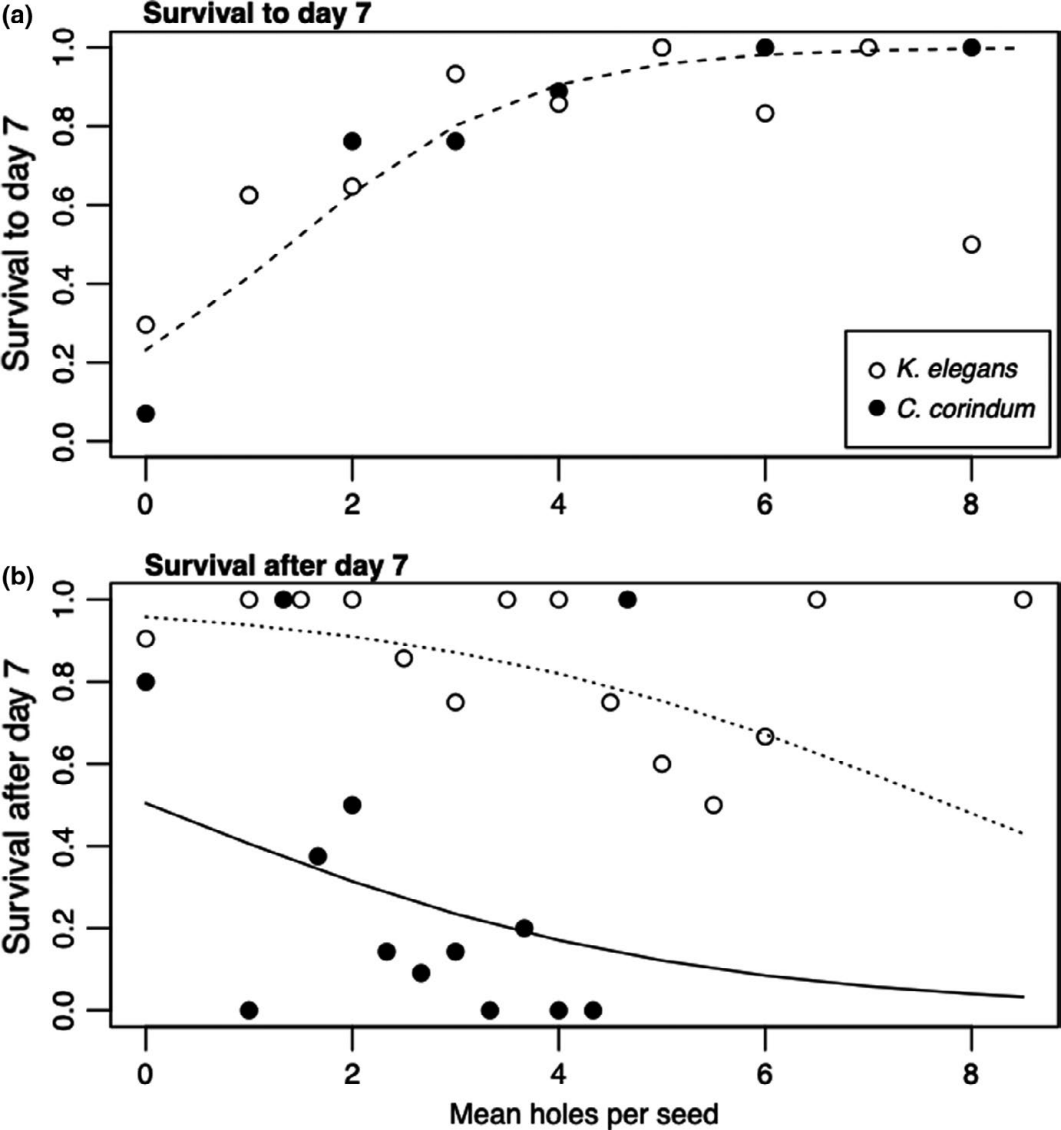
Survival prior to day 7 (a) and after day 7 (b) as a function of the number of holes drilled into treatment seeds by adults. All intact seeds have 0 holes. In (a), the dashed line shows model predictions for the relationship between hole number and survival up to day 7; this relationship did not differ between rearing hosts. In (b), the solid line shows model predictions for the relationship between hole number and survival after day 7 on rearing host *Cardiospermum corindum* and the dotted line shows model predictions on rearing host *Koelreuteria elegans*

Overall, juveniles in the prior feeding treatment were 2.4 times more likely to survive to adulthood than juveniles on the intact seed treatment (42.6% vs. 16.2%; *z*‐value = 5.18, *p* < 0.001). This effect was driven by facilitation early in development: Juveniles in the prior feeding treatment were 4.6 times more likely to survive the first week of development than juveniles in the intact seed treatment (78% vs. 18.3%; *z*‐value = 8.93; *p* < 0.001) (Figure 2a). Consistent with facilitation being the result of creating physical access points in the seed coat, there was a strong and positive effect of the number of holes drilled by adults in each seed on survival to day 7 in the prior feeding treatment (*z*‐value = 2.15, *p* =.03) (Figure 3a). These results were also found in prior feeding treatments with *K*. *paniculata* as the rearing host or field collection host (Appendices S1, S2; Figures S1, S4).

### 3.2 (H 2) Late in development, prior adult feeding has strong negative effects on juvenile performance

In contrast, the effects of the prior feeding treatment later in development were no longer positive. Bugs reared in the prior feeding treatment were 2.3 times as likely to die late in development as juveniles in the intact seed treatment (54.5% vs. 88.5%; *z*‐value = 2.17, *p* = 0.03) (Figure 2c). Increasing the number of holes within the prior feeding treatment had a strong negative effect on survival after day 7 (*z*‐value = −2.27, *p* = 0.02) (Figure 3b).

**FIGURE 4:**
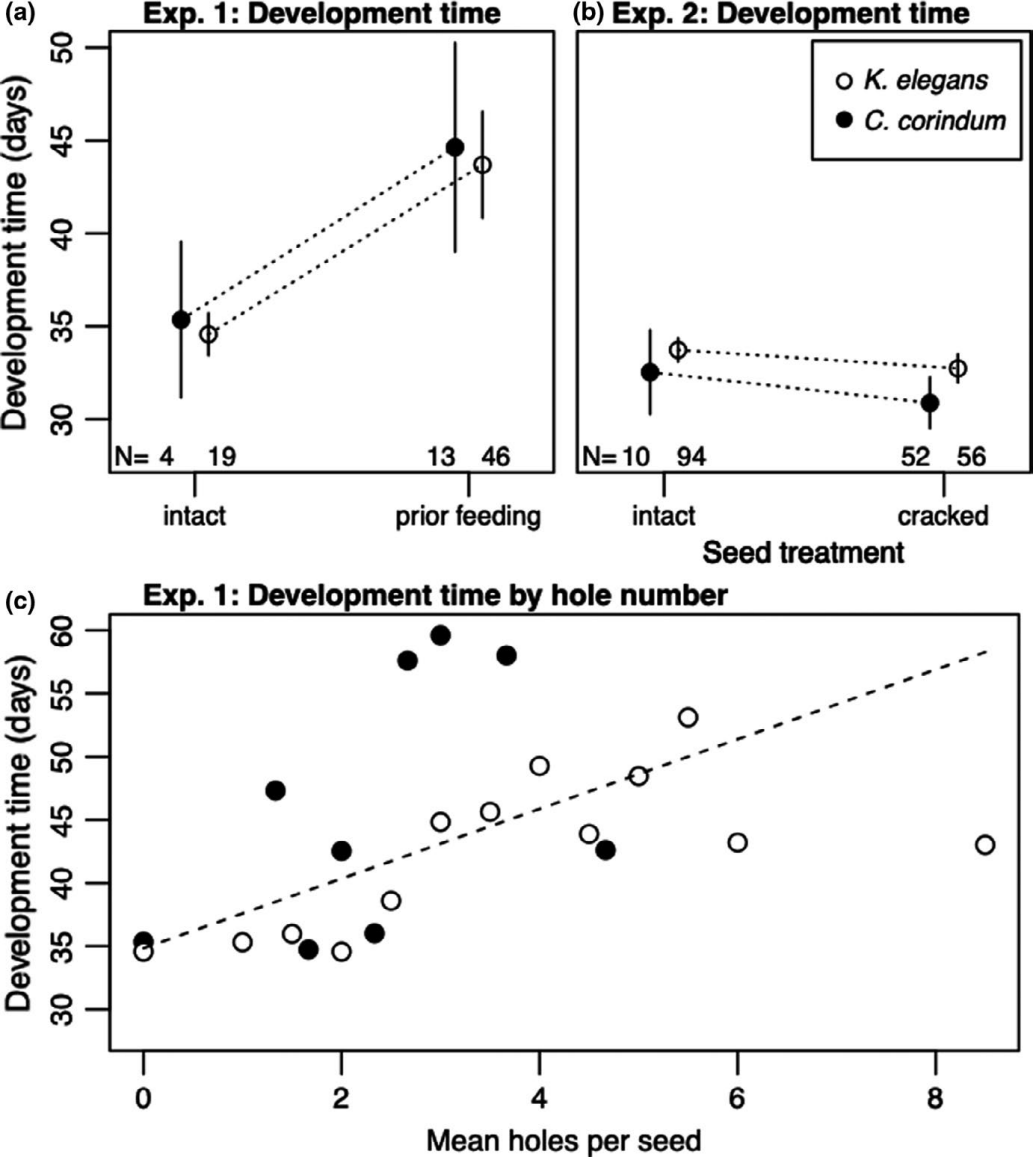
Development time from hatching to adulthood. All development times are corrected for field collection host and sex. (a) Mean development times in Experiment 1 for bugs reared on intact seeds and seeds with prior feeding from *Cardiospermum corindum* and *Koelreuteria elegans*. (b) Mean development times in Experiment 2 for bugs reared on intact and cracked seeds of *C*. *corindum* and *K*. *elegans*. (c) Development time as a function of the number of holes in each seed for bugs reared on *C*. *corindum* and *K*. *elegans* in Experiment 1. The dashed line shows model predictions for the relationship between hole number and development time, which did not differ detectably between rearing hosts

The prior feeding treatment also increased the amount of time it took for juveniles to reach adulthood by 4.44 ± 0.90 days (*t*‐value = −4.18, *p* < 0.001, *df* = 78) (Figure 4a). Within the prior feeding treatment, each additional hole created by adult feeding increased development time by 3.30 ± 0.91 days (*t*‐value = 3.60, *p* < 0.001, *df* = 56) (Figure 4c).

**FIGURE 5:**
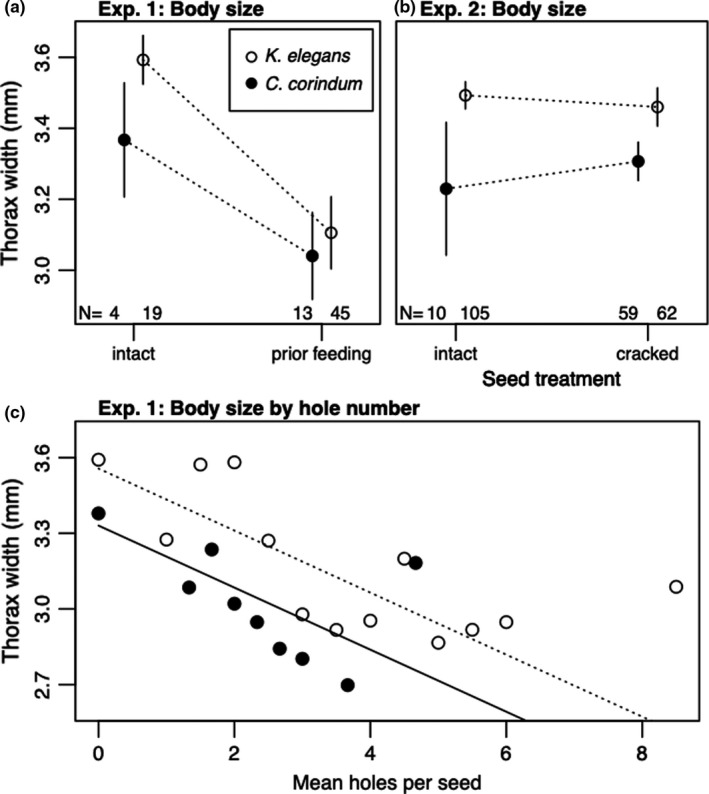
Adult thorax width by treatment. All measures are corrected for sex. (a) Mean adult thorax width (mm) in Experiment 1 on rearing hosts *Cardiospermum corindum* and *Koelreuteria elegans* on intact seeds and seeds with prior feeding. (b) Mean adult thorax width (mm) in Experiment 2 on rearing hosts *C*. *corindum* and *K*. *elegans* on intact and cracked seeds. Points represent treatment means, and error bars represent 95% confidence intervals. (c) Thorax width as a function of the number of holes in each seed. Lines represent model predictions from the top model for *C*. *corindum* (solid line) and *K*. *elegans* (dotted line)

Adults reared in the prior feeding treatment were significantly smaller than those in the intact treatment, with thorax width reduced by 0.23 ± 0.04 mm (*t*‐value = −6.64, *p* < 0.001, *df* = 76) (Figure 5a). Within the prior feeding treatment, each additional hole in a seed decreased adult thorax width by 0.09 ± 0.03 mm (*t*‐value = −3.12, *p* = 0.003, *df* = 55) (Figure 5c).

Within the *K*. *paniculata* field collection host and rearing host treatments (Appendices S1 and S2), I found similar results for the impacts of prior feeding on early and late survival (Figures S1, S4), development time (Figures S2, S5), and body size (Figures S3, S6).

### 3.3 (H 3) Cracking the seed coat increases juvenile survival

In Experiment 2, the cracked seed treatment mimicked the positive effects of adult feeding on juvenile survival, producing a dramatic increase in survival probability such that the odds of individuals in the cracked treatment surviving to adulthood were 6.9 times those of individuals in the intact treatment (96.5% vs. 45.6%; *z*‐value = 6.21, *p* < 0.001).

This was again largely due to positive effects in the first week of development (when 94% of mortality occurred): the odds of an individual in the cracked treatment surviving to day 7 increased by 7.6 compared to individuals in the intact treatment (98.2% vs. 49.6%; *z*‐value = 5.50, *p* < 0.001) (Figure 2b).

Unlike the prior feeding treatment in Experiment 1, the cracked treatment had a positive or neutral effect on fitness later in development. The odds of a juvenile in the cracked seed treatment surviving late in development increased by 4.3 in the cracked versus the intact seed treatment (92% vs. 98.2%; *z*‐value = 3.19, *p* = 0.001) (Figure 2d). On average, juveniles in the cracked seed coat treatment developed 0.8 ± 0.3 days more quickly than those on the intact treatment (*t*‐value = −1.97, *p* = 0.05, *df* = 207) (Figure 4b). Juveniles in the cracked and intact seed treatments did not differ in their final adult body size (Figure 5b).

### 3.4 (H 4) Juveniles reared on the native host experience lower fitness and greater benefits of facilitation than those on the introduced host

The rearing host *C*. *corindum* generally had stronger negative effects on performance than the introduced *K*. *elegans*. In both experiments, individuals who developed on *C*. *corindum* had lower survival both early (Exp 1: *z*‐value = −2.90, *p* = 0.004; Exp 2: *z*‐value = −2.50, *p* = 0.012; Appendix S1) and late (Exp 1: *z*‐value = −2.61, *p* = 0.009; Exp 2: *z*‐value = −3.53, *p* < 0.001). They also generally achieved smaller adult body sizes, although this was only significant in Experiment 2 (Exp 1: *t*‐value = −1.65, *p* = 0.10, *df* = 76; Exp 2: *t*‐value = −5.55, *p* < 0.001, *df* = 207). The effects of rearing host plant on development time were mixed, with *C*. *corindum* causing no significant effect on development time in Experiment 1 (*t*‐value = 0.38, *p* = 0.7, *df* = 78) and faster development times in Experiment 2 (*t*‐value = −2.64, *p* = 0.009, *df* = 207).

Prior feeding by adults had a stronger positive effect on early survival on the native rearing host than on the introduced rearing host (host * seed treatment interaction: *z*‐value = 2.21, *p* = 0.027). These effects were paralleled in Experiment 2: The improvements in early juvenile survival caused by cracking the seed coat were stronger when juveniles were reared on the native host than on the invasive host (host * seed treatment interaction: *z*‐value = 2.50, *p* = 0.012).

There were no interactions found between treatments and field collection host where tested. However, bugs from field collection host *C*. *corindum* generally had lower survival (Experiment 1:19.8% vs. 36.9%; *z*‐value = −3.48; *p* = 0.0005), especially late in ontogeny (Experiment 1:45.5% vs. 71.6%; *z*‐value = −3.26; *p* = 0.001; Experiment 2:90.4% vs. 96.9%; *z*‐value = −2.1; *p* = 0.036). Bugs from the native field collection host *C*. *corindum* also had significantly longer development times than those from the introduced field collection host *K*. *elegans* (Experiment 1:46.12 days vs. 36.61 days; *t*‐value = 4.051; *p* = 0.0001; Experiment 2:35.04 days vs. 31.41 days; *t*‐value = 5.2; *p* < 0.0001).

## 4 DISCUSSION

I found strong support for my hypothesis that prior adult feeding facilitates early juvenile survival. Consistent with physical access through the seed coat limiting early juvenile success, the number of holes produced by adult feeding was a strong predictor of early survival, an effect that was mimicked by manually cracking the seed coat. After the vulnerable first week of development, however, the effects of prior adult feeding became strongly negative, an effect that was not produced by manual cracking. Thus, early facilitation came at a cost to later developmental stages, potentially due to direct competition for resources. I also found that bugs reared on the native *C*. *corindum*, which was a more stressful rearing host, were more sensitive to the positive effects of early adult facilitation than those reared on the introduced *K*. *elegans*.

I find that facilitation is key early in development for juvenile soapberry bugs and is highly correlated with the number of holes drilled by adults through the seed coat on both host plants. Furthermore, the strength of this positive effect was greater on the native host, which has seeds that are very difficult to penetrate. This positive effect can be reproduced by manually cracking the seed coat in the absence of prior adult feeding. All of this evidence indicates that the seed coat is the primary driver of early juvenile mortality, especially on the native host plant. However, it may not be the only important factor for early juvenile survival—as in many plant‐feeding insects, chemical defenses may play an important role. If defensive compounds are part of the seed coat itself, cracking the seed coat would also allow juveniles to circumvent this barrier.

Late in development, prior adult feeding has strong negative effects on juvenile fitness. The amount of damage created by adults (hole number), which had strong positive effects in early development, becomes negative for older juveniles. This is not due to direct negative effects of breaking through the seed coat (e.g., through drying out of seed contents), because cracking the seed coat manually has positive effects for older juveniles. Increased late mortality, prolonged development times, and smaller body sizes are all consistent with nutrient deprivation following the loss of resources to adults. They may also be explained by adults altering the nutritional or toxin content of the seeds. When juvenile and adult soapberry bugs feed, they inject digestive compounds into the seed. It is not clear a priori whether the impacts of adult digestive juices would be positive or negative for juvenile fitness (e.g., they could predigest seed contents, making juvenile feeding easier); however, the combined results of both of these experiments suggest they are negative. Early juvenile survival was about 20% lower in the prior feeding treatment of Experiment 1 (78%) than in the cracked treatment of Experiment 2 (98%), suggesting that adults breaking the seed coat barrier come with immediate negative consequences, even for first instar juveniles feeding after adults. These differences should be taken with a grain of salt, as these two experiments differed in the total survival within the intact treatment; however, there are several plausible biological explanations for this pattern that warrant further exploration. Adult digestive juices, or non‐self‐digestive juices generally, might contain compounds that have a degree of toxicity for feeding juveniles. They could also accelerate spoilage of seed contents or induce a chemical defense in the remaining plant within the seed. Further experiments are needed to distinguish these mechanisms.

The difference between experiments in the intact treatment is striking enough to warrant further exploration. The main difference in experimental methods for the intact treatment was that seeds in Experiment 1 were soaked in water for a prolonged period (18 hr) and held at 28℃, while those in Experiment 2 were only tested for viability in water briefly and held at room temperature prior to juvenile feeding. Seeds in Experiment 1 were treated this way for consistency with the adult feeding treatment, but these same factors that motivated adult feeding may have directly negatively impacted juvenile fitness. Soaking and/or elevated temperatures could have caused the seed coat to take on water and expand, widening the barrier between soapberry bug and developing plant. Water is a germination cue for many plant seeds, and while we do not know what the chemical responses are in these species, could potentially have caused the developing plant to express defensive compounds that reduced overall survival across treatments. Because experiments were conducted in different years and were unlikely to be identical in every way, I cannot say definitively that this different treatment of seeds was the driving factor for the difference in mortality between experiments. However, this does suggest that seed defenses are plastic and future investigations quantifying the effect of environmental conditions on seed defenses and nutrition would be fruitful.

The final major result of this experiment was the stronger positive impact of facilitation for juveniles feeding on the native host (*C*. *corindum*) than for those on the introduced host (*K*. *elegans*). Juveniles reared on the native host had lower survival across the board than those reared on the introduced host. If we relate this back to the Stress Gradient Hypothesis in plants, the native host is akin to harsh growing environments, both through extreme early mortality (Figure 2) and low reproduction (Cenzer, 2017), while the introduced host has much weaker negative effects on both. Consistent with the Stress Gradient Hypothesis, the benefits of breaking through the seed coat were stronger on the more stressful *C*. *corindum* than on the more palatable *K*. *elegans*. Indeed, the positive effects of early facilitation on *C*. *corindum* so far outstripped those on *K*. *elegans* that they equalized early survival, making the harsh environment just as benign as the palatable one. This was true both for manual cracking of the seed coat and for actual facilitation by adult conspecifics. While the difference in the toughness of the seed coat for these two plant species is unambiguous, species of soapberry bugs occupy a huge range of host plants throughout their global range (Carroll & Loye, 2012). Exploring the generality of the impacts of the seed coat across a broader range of host species, particularly with more quantifiable metrics of seed coat toughness, is a valuable direction for future research.

If we consider harsher environments in a temporal, rather than spatial, framework, then life stages that experience greater fitness costs when developing alone may also gain greater benefits from facilitation. Thus, for organisms who experience high mortality early and low mortality late in development when raised alone, we should predict a shift from facilitation to competition when they occur with conspecifics. This pattern of shifts from facilitation to competition is thought to be common for plants growing in extreme environments, where “nurse plants” create favorable local abiotic conditions for vulnerable seedlings which later become competitors. These ontogenetic shifts in plants have been documented through spatial associations (e.g., le Roux et al., 2013; Valiente‐Banuet & Verdu, 2008), long‐term observations of natural recruitment and performance (e.g., Miriti, 2006), and a handful of experimental manipulations (Loranger et al., 2017; Schiffers & Tielborger, 2006). Although the system and environmental stressors are different, for young juvenile soapberry bugs, the seed coat defense has extremely negative effects on fitness which decrease in severity with age; in parallel, we see the relative benefits of facilitation decrease over soapberry bug ontogeny.

This ontogenetic shift may create a conflict between developmental stages, as older juveniles make seeds accessible to younger juveniles, and bear the cost of competition without the benefits of facilitation. Very young juveniles should optimally seek out seeds with prior feeding while older juveniles should feed on pristine seeds, promoting behavioral changes across ontogeny from aggregative to more solitary. This pattern of behavior has been observed in other insect herbivores with synchronous development (e.g., eggs from a single clutch: Denno & Benrey, 1997; Fordyce, 2003; Inouye & Johnson, 2005). The existence of a life stage that relies on facilitation could promote the maintenance of social behavior, even when individuals are not related, at least until individuals are capable of dispersing away from their natal habitat.

This study highlights the potential for transitions from facilitation to competition as a potentially important pattern for systems where stress tolerance varies across ontogeny. I find support for the hypothesis that early facilitation is more likely in harsher environments, in this case, on the more well‐defended host plant, demonstrating that the relative importance of facilitation and competition varies both across age and environment. Further studies of ontogenetic shifts from facilitation to competition and their mechanisms, particularly in animal systems, are needed to better understand the generality of these results.

## CONFLICT OF INTEREST

MLC: Conceiving, designing, execution of this study and writing the manuscript. No other person is entitled to authorship. There are no conflicts of interest.

## AUTHOR CONTRIBUTION

**Meredith Cenzer:** Conceptualization (equal); Data curation (equal); Formal analysis (equal); Funding acquisition (equal); Investigation (equal); Methodology (equal); Project administration (equal).

## Supporting information

Appendix S1‐S2

## Data Availability

Associated data are publicly available on Dryad: https://doi.org/10.5061/dryad.34tmpg4ks.
