# Supplementary material for: Adult facilitation becomes competition as juvenile soapberry bugs age
Source: Ecol Evol. 2021 Sep 8;11(19):13335–47. doi: 10.1002/ece3.8056 (PMC8495836; doi:10.1002/ece3.8056)
Supplement: Supplementary file 1 — Appendix S1‐S2 [file ECE3-11-13335-s001.docx]

**Appendix S1**

In the 1990s, soapberry bugs were unintentionally introduced to Davis, California where their primary host is *K. paniculata*, a second introduced species of golden rain tree. There have been no formal studies yet of adaptations to this host in California. Experiment 1 described in the main text was also performed on the introduced golden rain tree species *Koelreuteria paniculata*, in Davis, CA, as both an ancestral host and a rearing host. Similar to *K. elegans*, this host has a single mass fruiting event each year with seeds persisting for several months before bugs must diapause over the summer. Seedpods are also open during the development of the seed. Seeds of *K. paniculata* are slightly larger than those of *K. elegans*. To the best of my knowledge, this host is also not consumed by any other insects in its introduced range.

BUGS FROM ANCESTRAL HOST *K. PANICULATA* SHOW THE SAME PATTERNS AS THOSE FROM BOTH OTHER ANCESTRAL HOSTS.

Total survival for bugs from ancestral host *K. paniculata* was higher on *K. elegans* than on *C. corindum* (z-value=3.91; p<0.001). There was not a significant effect of prior feeding on total survival (z-value=1.15; p=0.25). There was a strong positive effect of prior feeding on survival early in development (z-value=3.75; p<0.001)(Fig. S1a); within the prior feeding treatment, there was a positive effect of the number of holes drilled in the seed with increasing survival (z-value=2.34; p=0.02)(Fig. S1b). The trend was negative, but there was not a significant direct effect of treatment or hole number on survival late in development (Figs. S1c & S1d).

The prior feeding treatment increased overall development time (t-value=2.75, p=0.012), with a marginally stronger effect for females than for males (t-value=1.77, p=0.09, df=20)(Fig. S2). The prior feeding treatment also significantly reduced body size (t=-3.19, p=0.005, df=20)(Fig. S3).

There was not a detectable effect of hole number on development time or body size; however, sample sizes were very low for this group, so subtle effects were probably not detectable.

Figure S1: Early (a, b) and late (c, d) survival for soapberry bugs from ancestral host *K. paniculata* when reared on *C. corindum* and *K. elegans*. Only one individual survived early development in order to have a late survival (c) in the intact *C. corindum* treatment; therefore, this point has no confidence interval plotted.

Figure S2: Development time in days for soapberry bugs from the ancestral host *K. paniculata* reared on *C. corindum* and *K. elegans* in Experiment 1. Only one individual survived to adulthood in the intact *C. corindum* treatment; therefore, this point has no confidence interval plotted.

Figure S3: Body size for soapberry bugs from the ancestral host *K. paniculata* when reared on *C. corindum* and *K. elegans* in Experiment 1. Only one individual survived to adulthood in the intact *C. corindum* treatment; therefore, this point has no confidence interval plotted.

**Appendix S2**

TREATMENT EFFECTS ARE CONSISTENT FOR BUGS ON REARING HOST *K. PANICULATA*

The prior feeding treatment had a strong positive effect on total survival for bugs reared on *K. paniculata* (z-value=5.32; p<0.001). There was a very strong positive effect of prior feeding on early survival (z-value=5.98; p<0.001)(Fig. S4a). Increasing the number of holes in the seed had a strong relationship with early survival (z-value=4.22; p<0.001)(Fig. S4b). There was no effect of the treatment on late survival (Fig. S4c). Individuals from ancestral host *K. elegans* had lower late survival than those from *C. corindum* when reared on *K. paniculata* (z-value=-2.22; p=0.026). There was no significant effect of increasing hole number on late survival on rearing host *K. paniculata* (Fig. S4d).

There was a marginally significant effect of the prior feeding treatment increasing development time when reared on *K. paniculata* (t-value=-1.88, p=0.068, df=43)(Fig. S5). There was not a detectable effect of the number of holes on increasing development time on this host (t-value=0.93; p=0.36). There was no significant effect of treatment or ancestral host on body size (Fig. S6a); however, as the number of holes increased, body size did show a marginally significant decrease (t-value=-1.93; p=0.06; df=38)(Fig. S6b).

Figure S4: Early (a, b) and late (c, d) survival for soapberry bugs reared on *K. paniculata* from ancestral hosts *C. corindum*, *K. elegans*, and *K. paniculata*.

Figure S5: Development time in days for soapberry bugs reared on *K. paniculata* from ancestral hosts *C. corindum*, *K. elegans*, and *K. paniculata* in Experiment 2. Only one individual survived to adulthood in the intact *C. corindum* treatment; therefore, this point has no confidence interval plotted.

Figure S6: Body size for soapberry bugs from the ancestral host reared on *K. paniculata* from ancestral hosts *C. corindum*, *K. elegans*, and *K. paniculata* in Experiment 2. a. Body size in each seed treatment. b. Body size as a function of hole number. Only one individual survived to adulthood in the intact *C. corindum* treatment in order to have a body size measure (a); therefore, this point has no confidence interval plotted.
